# Presepsin as Early Marker of Sepsis in Emergency Department: A Narrative Review

**DOI:** 10.3390/medicina57080770

**Published:** 2021-07-29

**Authors:** Andrea Piccioni, Michele Cosimo Santoro, Tommaso de Cunzo, Gianluca Tullo, Sara Cicchinelli, Angela Saviano, Federico Valletta, Marco Maria Pascale, Marcello Candelli, Marcello Covino, Francesco Franceschi

**Affiliations:** 1 Emergency Medicine Fondazione Policlinico Universitario A. Gemelli IRCCS, 00168 Rome, Italy; andrea.piccioni@policlinicogemelli.it (A.P.); sara.cicchinelli@policlinicogemelli.it (S.C.); marcomaria.pascale@policlinicogemelli.it (M.M.P.); marcello.candelli@policlinicogemelli.it (M.C.); marcello.covino@policlinicogemelli.it (M.C.); francesco.franceschi@policlinicogemelli.it (F.F.); 2 Fondazione Policlinico Universitario A. Gemelli IRCCS, Università Cattolica del Sacro Cuore, 00168 Rome, Italy; tomdecunzo@gmail.com (T.d.C.); gianlucatullo@gmail.com (G.T.); saviange@libero.it (A.S.); fede.valletta@gemelli.com (F.V.)

**Keywords:** Presepsin, sepsis, emergency department, critical care, ICU

## Abstract

The diagnosis and treatment of sepsis have always been a challenge for the physician, especially in critical care setting such as emergency department (ED), and currently sepsis remains one of the major causes of mortality. Although the traditional definition of sepsis based on systemic inflammatory response syndrome (SIRS) criteria changed in 2016, replaced by the new criteria of SEPSIS-3 based on organ failure evaluation, early identification and consequent early appropriated therapy remain the primary goal of sepsis treatment. Unfortunately, currently there is a lack of a foolproof system for making early sepsis diagnosis because conventional diagnostic tools like cultures take a long time and are often burdened with false negatives, while molecular techniques require specific equipment and have high costs. In this context, biomarkers, such as C-Reactive Protein (CRP) and Procalcitonin (PCT), are very useful tools to distinguish between normal and pathological conditions, graduate the disease severity, guide treatment, monitor therapeutic responses and predict prognosis. Among the new emerging biomarkers of sepsis, Presepsin (P-SEP) appears to be the most promising. Several studies have shown that P-SEP plasma levels increase during bacterial sepsis and decline in response to appropriate therapy, with sensitivity and specificity values comparable to those of PCT. In neonatal sepsis, P-SEP compared to PCT has been shown to be more effective in diagnosing and guiding therapy. Since in sepsis the P-SEP plasma levels increase before those of PCT and since the current methods available allow measurement of P-SEP plasma levels within 17 min, P-SEP appears a sepsis biomarker particularly suited to the emergency department and critical care.

## 1. Introduction

The diagnosis and treatment of sepsis have always been a challenge for the physician, especially in critical care setting. Indeed, sepsis is one of the major causes of mortality in both emergency department (ED) and intensive care unit (ICU), due to main difficulty of early recognition and appropriate identification of the etiology [1,2,3]. In many cases, infections are characterized by signs and symptoms that can overlap with other acute disease, therefore differential diagnosis is crucial, but often demanding, and leads to a double issue. On the one hand, the untimely identification of a sepsis leads to a therapeutic delay with a consequent increase in mortality; on the other hand, often patients are treated with unnecessary antibiotic therapy, which is one of the main causes of antibiotic resistance [4,5,6]. The traditional definition of sepsis, since 1992 referred to as the presence or suspected infection associated with a systemic inflammatory response syndrome (SIRS) [7], changed in 2016, replaced by the new criteria of SEPSIS-3 [8], so that sepsis is currently defined as infection with organ dysfunction, assessed by the Sequential Organ Failure Assessment (SOFA score), while the previous expression “severe sepsis” is no longer adopted to increase predictive accuracy [9]; however, early identification and consequent early appropriated therapy remain a cornerstone of sepsis treatment. In clinical practice the diagnosis of sepsis is based on tools such as cultures, which take a long time and are often burdened with false negatives, while the use of molecular techniques requires specific equipment and skilled operators, thus entailing very high costs [5,10]. Therefore, currently there is a lack of a foolproof system for making early sepsis diagnosis. In this context, biomarkers, defined as objectively measurable characteristics of biological processes, are very useful tools to distinguish between normal and pathological conditions, graduate the disease severity, guide treatment, monitor therapeutic responses and predict prognosis [11,12]. Alongside the more widely spread and employed markers, such as C-Reactive Protein (CRP) and Procalcitonin (PCT), there are new ones emerging, among which P-SEP appears to be the most promising [13]. The purpose of this review is to search, by consulting electronic databases, the main studies performed in the last 10 years concerning the use of P-SEP in sepsis, with particular attention to those concerning the usefulness of P-SEP in the emergency department.

## 2. Methods and Results

We checked medical literature of the last 10 years to find P-SEP related studies and reports. The following electronic databases were systematically searched: MEDLINE-PubMed, Web of Science, Scopus, and the Cochrane Central Register of Controlled Trials (CENTRAL). The search strings were:

- Presepsin AND sepsis
- Presepsin AND emergency department
- Presepsin AND critical care
- Presepsin AND ICU

The search strategy was limited to English language articles. We mainly focused on randomized placebo-control studies, followed by case-control studies, observational (both retrospective or prospective), and finally systematic reviews and meta-analysis. The article selection process was carried out independently by two reviewers (MCS and TdC). Additional manual scrutiny carried out from the references of the selected articles was performed in order to identify other potentially relevant studies.

We identified a total of 224 studies deemed to be relevant for the issues in stake (Table 1). Among these, 166 articles are clinical trials, of which 4 are randomized controlled trials. The remaining articles consist in 38 reviews, and 13 systematic reviews and meta-analysis (Table 2). As summarized in Table 2, the meta-analysis performed in 2015 mainly focused on validating the diagnostic value of P-SEP in sepsis, while the meta-analysis conducted between 2016 and 2019 analyzed the prognostic value of P-SEP in comparison and in combination with other biomarkers of sepsis, especially the more common used C-RP and PCT [14,15,16,17,18,19,20,21,22,23,24,25,26].

## 3. Current Data on Presepsin as a Biomarker for Sepsis

### 3.1. Presepsin

**Figure 1:**
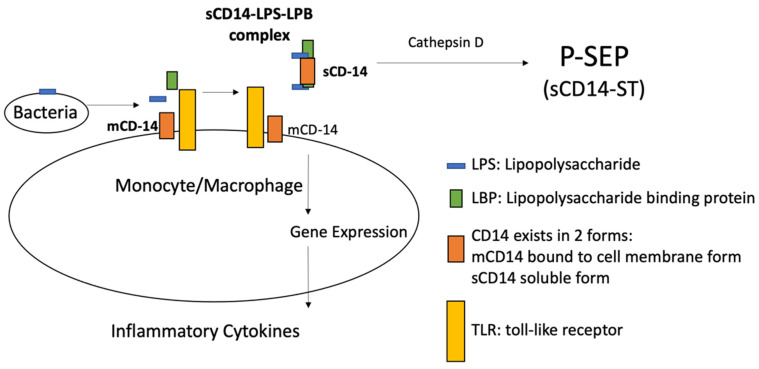
P-SEP is the 13 KDa N-terminal fragment of soluble form of CD14 (sCD14), cleaved by cathepsin D in plasma, and involved in activating the innate immune system.

P-SEP is the subtype of the soluble form of CD14 (sCD14-ST); more precisely, P-SEP is the 13 KDa N-terminal fragment of soluble form of CD14 (sCD14), cleaved by cathepsin D in plasma, and involved in activating the innate immune system. [27]. Especially in the last decade, several studies have shown increases in response to bacterial infections and decreases after healing or effective treatment [6,14], so that P-SEP is considered a new biomarker, effective in early recognition of different types of infections [28,29]. As known, infections activate the host’s immune system, usually distinguished into innate and an adaptive: while adaptive needs several days to be effective, an innate system provides an immediate response mainly through the alternative complement system and phagocytosis [30,31,32]. Both systems need to recognize the pathogens, but the innate one carries out recognition through different receptors, that are already predetermined, placed on the immune effector cell surface [33,34], able to recognizing a wide range of antigens on the surface of most microbial pathogens [35]. CD14 is 55 KDa transmembrane glycoprotein acting as a coreceptor, placed on the monocytes and macrophages cell surface, belonging to the family of Toll-like receptors (TLRs), which play a role in the identification of several Gram-positive and Gram-negative bacterial ligands [31,36]. In particular, the recognition of Lipopolysaccharide (LPS), present on the Gram-negative bacteria surface, requires the association of the Lipoprotein Binding Protein (LBP), which presents the LPS at CD14; the CD14-LPS-LBP complex stimulates intra-cellular signals that promote the expression of genes involved in the immune response such as cytokines production by effector cells [37]. CD14 exists in two forms: one bound to the membrane (mCD14) of monocyte and macrophage cells, and a soluble one (sCD14) present in the plasma, where it is cleaved by cathepsin D into a fragment of 13 kDa (sCD14-ST), precisely named, released in the general circulation by proteolysis and exocytosis [13,37] (Figure 1).

### 3.2. Presepsin Measurement

Since P-SEP is released during the activation of the immune system, it is essential to have a rapid and accurate method to measure P-SEP plasma level and establish a cut-off, which allows distinguishing sick individuals from healthy ones [38]. The method initially developed was a conventional two-step ELISA assay, measuring P-SEP in a range of 3–150 ng/mL, but requiring a total assay time of 4 h and most importantly showing low accuracy. Subsequently, producing a one-step instead of a two-step method and using recombinant P-SEP instead of recombinant CD14, Thermo Fisher developed a faster ELISA, resulting in a reduction of the total assay time from 4 to 1.5 h and a change in range of 0.05–3.00 ng/mL (or 50–3000 ng/L) instead of 3-150 ng/mL (obtained with the previous two-step ELISA) [39]. Based on chemiluminescence enzyme immunoassay (CLEIA) in combination with MAGTRATION technology, PATHFAST (Mitsubishi Chemical) is an innovative, highly sensitive and fully automated method, which allows the measurement of P-SEP plasma levels, using whole blood samples, able to provide results within 17 min in six samples simultaneously [40,41]. This feature, together with the non-interference by other analytes such as hemoglobin, bilirubin, lipids, makes PATHFAST a very useful tool in critical areas, especially in an emergency department, where rapid quantitative results are required.

### 3.3. Diagnostic Significance of Presepsin in Sepsis

The results of several prospective multicenter trials, manly conducted in the last decade, have shown that, using a cut-off value of 600 ng/L, P-SEP plasma levels are significantly elevated in patients with bacterial infection compared to non-bacterial infections, with sensitivity and specificity of 87.8% and 81.4%, respectively [42,43,44,45]. Another study has shown that using a cut off of 670, sensitivity and specificity were reported to be 70.3% and 81.3%, respectively, while at a cut off value of 864 ng/L, sensitivity and specificities were 71.4% and 63.8%, respectively [46]. Several studies reported that a cut off of 600 ng/mL is not able to discriminate between Gram positive and Gram-negative and was not related to the positivity or negativity of blood cultures, while in 2015 in the multicentric randomized trial ALBIOS Masson et al. found that at a baseline concentration of 946 ng/L plasma P-SEP levels are higher in patients with Gram-negative than in Gram-positive bacterial infections [47]; it is hypothesized that this is due to the role played by P-SEP in the formation of the CD14-LPS-LBP complex. The ALBIOS trial also reported that the P-SEP value (mean ± standard deviation), in the healthy, SIRS and sepsis group, was 258.7 ± 92.53 ng/L, 430.0 ± 141.33 ng/L and 1508.3 ± 866.6 ng/L respectively [47]. However, in a 2015 meta-analysis, Wu et al. reported that P-SEP has only moderate diagnostic accuracy in differentiating sepsis from other non-septic inflammatory conditions, suggesting that the results should be interpreted with caution and further studies are needed before considering P-SEP as a definitive marker for the diagnosis of sepsis [15].

### 3.4. Prognostic Significance of Presepsin in Sepsis

The change in P-SEP plasma levels is a solid prognostic and therapeutic tool in hospitalized patients, however the P-SEP measured on arrival in the emergency department can be useful in risk stratification [48,49], especially for Gram-negative bacterial infection, probably due to the sCD14-ST role in the sepsis cascade as a receptor for LPS [50]. In a 2018 meta-analysis, including 10 studies with a total of 1617 patients, Yang et al. reported that P-SEP plasma levels in the first sampling (within 24 h) was significantly lower among survivors than non-survivors (I^2^ = 79%, *p* < 0.01) [20], while in the subgroups, divided by severity of sepsis or infection site, P-SEP was consistently higher in non-survivors. In hospitalized patients, many studies show that in severe sepsis (according to the definition prior to SEPSIS-3) or septic shock, the reduction in P-SEP plasma levels is associated with an increased survival and indicates the effectiveness of the antibiotic therapy, with P-SEP tending to decline by day 7 in patients with positive blood cultures and appropriate antibiotic therapy. Speculatively, high P-SEP plasma levels in the seventh day is considered due to inappropriate or ineffective therapy even with positive blood cultures (mostly multidrug-resistant bacteria), and is associated with increased mortality, the onset of complications, such as prolonged need for ventilation and inotropic agents, therefore a prolonged length to stay in ICU, as well as the presence of acute or acute on chronic kidney injury. [46,51,52]. In a study conducted in ICU patients, P-SEP was effective in predicting sepsis with a sensitivity and specificity values of 84.6% and 62.5%, respectively, which were significantly related to APACHE II score (*p*-value = 0.016) [53].

### 3.5. Presepsin Compared to C-RP and PCT: Alone or in Company?

Recently, several studies have focused on the role of new and emerging biomarkers of sepsis, such as proadrenomedullin, interleukin-6 (IL-6), CD 64, the soluble form of triggering receptor expressed on myeloid cells-1 (sTREM-1) [54], however most of the studies were carried out comparing P-SEP with C-RP and PCT, which to date remain the most widely diffuse markers of sepsis in clinical practice [26]. The diagnostic and prognostic efficacy of P-SEP has been analyzed in different clinical settings not only as an alternative, but also in combination with C-RP and PCT; these comparative studies have reported controversial results [55,56,57,58,59]. In 2017, Kim et al. reported that, using a cutoff of 2455 ng/L, P-SEP is better than PCT in predicting mortality of sepsis at 30 days (AUC of 0.684 versus 0.513), being higher in non survivors than in survivors [55], while in another 2015 study, using a cut off of 413 ng/L for diagnosing bacterial infections in ICU patients, Godnic et al. reported that P-SEP has a higher AUC compared to PCT (0.705 vs. 0.630) but lower than C-RP (0.705 vs. 0.734) [53]. In 2016, Plesko et al. reported that in hematologic patients the association of P-ESP with IL-6 increases sensitivity compared to the use of P-SEP alone in detecting sepsis, while the association of P-SEP with PCT and C-RP did not show better accuracy than P-SEP alone in detecting sepsis in this type of patient [60]. Klouche et al. reported greater specificity of P-SEP and PCT in combination for the diagnosis of sepsis, septic shock and pneumonia, compared to using PCT alone or P-SEP alone [61].

### 3.6. Presepsin in Pediatric Bacterial Infection

Numerous studies have investigated the diagnostic and prognostic role of P-SEP in different type of infections in children, such as early (EOS) and late onset sepsis (LOS) in preterm infants, particularly meningitis and pneumonia, but also infections in febrile neutropenic patients affected by onco-hematological neoplasms [6,24,25,45,62,63,64,65,66,67,68]. In neonatal sepsis P-SEP compared to PCT has been shown to be more effective in diagnosing and guiding therapy [62]. In the EOS at the cutoff of 539 ng/L, P-SEP has showed a sensitivity of 80% and specificity of 75%, while in the LOS at the cutoff of 885 ng/L, P-SEP demonstrated a sensitivity of 94% and a specificity of 100% [21,22,69]. In the pediatric setting, mean P-SEP plasma levels in healthy infants are much higher (720 ng/L) than in healthy adults, probably due to the passage after birth from the intrauterine environment to the new external environment rich in foreign antigens, which activates the innate immune system [69].

### 3.7. Presepsin in Fungal Infection

Several recent studies have shown that fungi are responsible for about 20% of all cases of sepsis, with a fatal outcome reaching 80% [70]. For this reason, in recent years there has been a growing attention towards fungal sepsis, the differential diagnosis of which from bacterial sepsis is often very challenging since the clinical manifestations can be overlapped. The main issue of fungal infections concerns neutropenic patients suffering from onco-hematological neoplasms and immunosuppressed patients. In this category of patients, more recently in 2019, several studies have demonstrated the usefulness of P-SEP in combination with PCT and C-RP in the diagnosis of bacterial and fungal infections. Stoma et al. reported that in hematological patients undergoing stem cell transplantation a cutoff of 218 ng/L is indicative of bacteremia [71], while elevation of C-RP associated with plasma P-ESP in the normal range predicts a fungal infection in immunocompromised patients [50]. Other studies have confirmed that a fungal infection can be predicted by the combination of increased P-SEP plasma levels with little or no alteration in PCT [72] and that plasma PSP levels are related to the severity of sepsis [73].

### 3.8. Presepsin Significance in SARS-CoV-2 Infection

Some studies published in 2020 reported that P-SEP is effective in risk stratification in patients with SARS-CoV-2 pneumonia, but further studies are needed to solidify this assertion. In a case series of six patients with SARS-CoV-2 pneumonia, Fukada et al. found that elevated P-SEP plasma levels can predict evolution towards ARDS [74], and Zaninotto et al. confirmed the effectiveness the prognostic value of P-SEP in 75 patients with SARS-CoV-2 pneumonia admitted to infectious disease ward and ICU [75].

### 3.9. Presepsin in Emergency Department

The critical area setting, especially ED, is particularly suited to reveal the potential greater utility of P-SEP over PCT in the early diagnosis of sepsis. Several studies have shown that P-SEP has diagnostic and prognostic power substantially similar to PCT, but, unlike PCT, P-SEP increases earlier in bacterial infection and can be measured effectively and accurately within 17 min directly in the emergency department [53]. A 2013 prospective study, conducted on 859 consecutive ED patients with at least two SIRS criteria (as defined prior to SEPIS-3), showed that P-SEP plasma levels is useful both for the diagnosis and for prognosis of sepsis, since P-SEP has been shown to be effective in stratifying the severity of sepsis, septic shock and in predicting mortality at 28 days. This study showed that the sensitivity, specificity, positive predictive value (PPV), negative predictive value (NPV) and diagnostic accuracy vary according to the cutoff used for P-SEP plasma levels. Using a cutoff of 449 ng/L P-SEP grades the severity of sepsis with sensitivity of 82.4%, specificity of 72.4%, PPV of 71.3% and NPV of 83.2% with a predictive accuracy of 77.0%; using a cutoff of 550 ng/L P-SEP predicts septic shock with sensitivity, specificity, PVV and NPV of 85.7%, 63.6%, 28.5% and 96.3%, respectively, and a predictive accuracy of 66.8%; using a cutoff of 556 ng/mL P-SEP predicts mortality at 28 days with sensitivity of 62.2%, specificity of 66.8%, PPV of 48.3%, NPV of 78.0% and predictive accuracy of 65.3% [44]. In 2015, Carpio et al. performed another single-center prospective observational study, including 120 patients with SIRS or sepsis criteria (prior to SEPSIS-3) and 123 healthy controls, confirmed that P-SEP at a cutoff of 581 ng/L is effective in diagnosing sepsis, graduating severity of disease and differentiating between SIRS and sepsis in ED, with sensitivity of 61% and specificity of 100% [49]. Also in this study, as in the previous one, the performance of P-SEP varies according to the cutoff considered: using a cutoff of 273 ng/L, a sensitivity of 95.5% and specificity of 21.7% were found, while using a cutoff of 686 ng/L these values were 46.5% and 91.3%, respectively. A study performed by de Guadiana Romualdo et al. in 2014, including 226 patients admitted to the ED with SIRS criteria, of which 37 had positive blood culture (bacteremic SIRS group) and 189 had negative blood culture (non-bacteremic SIRS group), reported sensitivity, specificity, PPV and NVP values of 81.1%, 63%, 30% and 94.4%, respectively, for the diagnosis of SIRS using a cutoff of 729 ng/L [57]. In 2017 the same author examined a cohort of 223 admitted in ED for suspected sepsis using two different P-SEP cutoffs, 312 and 849 ng/L, and found sensitivity values of 97.1% and of 67.1% and specificity values of 16.9% and 80.8%, respectively [58]. It has been reported that, using a 101.6 ng/L cutoff, P-SEP, measured at the time of diagnosis in the ED 24 h before admission to the ICU, has values of sensitivity, specificity, PPV and NPV of 81.9%, 96.5%, 82.4% and 96.3%, respectively, thus allowing for better management in both severe sepsis and septic shock [22,59]. The different cutoff values reported in the different studies are likely due to heterogeneity regarding the clinical setting (ED, ICU), the sepsis criteria adopted (before or after SEPSIS-3) and the type of sample (plasma, serum or whole blood) for the measurement of the P-SEP.

### 3.10. Presepsin Use Caveat

There are several clinical conditions in which special care must be taken in interpreting altered P-SEP plasma levels [50]. The more common diagnostic limitation of P-SEP is likely renal failure. Since the kidney is involved in the P-SEP excretion, the P-SEP plasma levels are increased in patients with renal failure. For this reason, the cutoffs must be adapted in patients with chronic kidney disease and/or on hemodialysis treatment [76]. P-SEP is also affected by the translocation of intestinal microbial flora [77]. Some pathophysiological conditions, such as age (newborns and elderly individuals, especially if suffering from renal failure) or burns [78] can influence P-SEP plasma levels, which may be higher even in the absence of disease [76], while further investigations are needed to define the influence of steroid use on P-SEP [27]. All these conditions must be considered to avoid an incorrect diagnosis of sepsis and consequently inappropriate treatments.

## 4. Discussion and Conclusions

The diagnosis and treatment of sepsis have always been a challenge for the physician, especially in critical care setting such as emergency department, and currently sepsis remains one of the major causes of mortality. Although the traditional definition of sepsis based on systemic inflammatory response syndrome (SIRS) criteria changed in 2016, replaced by the new criteria of SEPSIS-3 based on organ failure evaluation, early identification and consequent early appropriated therapy remain the primary goal of sepsis treatment. Among the new emerging biomarkers of sepsis, P-SEP appears to be the most promising. The studies examined demonstrate that P-SEP is a valid and reliable biomarker of bacterial sepsis, especially Gram-negative bacteria, and it is also a tool effective in evaluating the efficacy of therapy since the P-SEP plasma levels decrease when therapy is effective and increase when therapy is ineffective. It also emerges that P-SEP has a diagnostic and prognostic power substantially comparable to PCT, even if not all authors agree on the diagnostic accuracy; many currently recommend not to use P-SEP alone, but in combination with other sepsis markers, as well as traditional diagnostic tools like cultures. Furthermore, in some clinical conditions, such as renal failure, P-SEP plasma levels can be altered in the absence of sepsis, so that different P-SEP cutoffs are reported. Studies performed in pediatric setting have shown that P-SEP is a more effective than PCT in diagnosing neonatal sepsis. In critical areas, in particular the emergency department, P-SEP appears to be the most promising sepsis marker, due to earlier plasma levels increase than PCT, and the currently available assays, which allow for obtaining the P-SEP plasma levels within 17 min, thus allowing an early recognition and therapy of sepsis already in ED. Further studies are needed to better define diagnostic cutoffs and better evaluate the diagnostic and prognostic utility of P-SEP compared to PCT in the emergency department.

## Figures and Tables

**Table 1:** List of studies.

Type	n°
Total studies	224
Review	38
Systematic review and meta-analysis	13
Clinical trial	162
Randomized controlled trial	4

**Table 2:** Systematic review and meta-analysis.

Author	Year	Patients	Results
Zhang, J. et al. [14]	2015	3052	P-SEP is an effective diagnostic marker for sepsis (DOR 18)
Wu, J. et al. [15]	2015	2159	P-SEP is an effective diagnostic marker for sepsis (DOR 21.73)
Zheng, Z. et al. [16]	2015	1757	P-SEP is an effective diagnostic marker for sepsis (DOR 14.25)
Tong, X. et al. [17]	2015	3109	P-SEP is an effective diagnostic marker for sepsis (DOR 21.56)
Liu, Y. et al. [18]	2016	10438	PCT, CRP, IL6, sTREM-1, P-SEP, LBP and CD64 have similar diagnostic accuracy in detecting sepsis (AUC 0.85, 0.77, 0.79, 0.85, 0.88, 0.71 and 0.96 respectively)
Wu, C.C. et al. [19]	2017	3470	P-SEP is a good predictor for sepsis (DOR 16) but there is no significant variations compared to PCT (14) or CRP (13)
Yang, H.S. et al. [20]	2018	1617	P-SEP can predict mortality in patients with sepsis (SMD survivors/non-survivors 0.92)
Ruan, L. et al. [21]	2018	2661	Combination of PCT and CRP (DOR 79) or P-SEP alone (864) have better diagnostic power than CRP (19) and PCT (31) alone in neonatal sepsis
Yoon, S.H. et al. [22]	2019	308	P-SEP has higher diagnostic accuracy than PCT or CRP in detecting sepsis in children (OR 32.87, 11.8 and 4.63 respectively)
Kondo, Y. et al. [23]	2019	3012	Diagnostic accuracy in detecting infection is similar for PCT and P-SEP (sensibility 0.80 and 0.84, specificity 0.75 and 0.73 respectively)
Parri, N. et al. [24]	2019	636	Diagnostic accuracy of P-SEP resulted high in detecting neonatal sepsis (DOR 120.94)
van Maldeghem, I. et al. [25]	2019	1369	P-SEP is an effective diagnostic marker for sepsis in neonates (AUC 0.9639)
Zhu, Y. et al. [26]	2019	1561	PCT and P-SEP are both an effective diagnostic marker for sepsis (DOR 10 and 9 respectively)

## Data Availability

Not applicable.
